# Brain morphology in the peracarid crustacean *Neomysis integer* (Leach, 1814) with an emphasis on sexual dimorphism of the olfactory pathway

**DOI:** 10.1007/s00441-025-03978-y

**Published:** 2025-05-14

**Authors:** Katja Kümmerlen, Johanna Blatt, Lena Hoffmann, Steffen Harzsch

**Affiliations:** Department of Cytology and Evolutionary Biology, Zoological Institute and Museum, University of Greifswald, Soldmannstrasse 23, D.-17489 Greifswald, Germany

**Keywords:** Chemical senses, Setae, Aesthetasc, Neurochemistry, Amphipoda

## Abstract

Our current understanding of brain organization in malacostracan crustaceans is strongly biased towards representatives of the Decapoda (“ten legged” crustaceans) such as crayfish, crabs, clawed lobsters and spiny lobsters. However, to understand aspects of brain evolution in crustaceans, a broader taxonomic sampling is essential. The peracarid crustaceans are a species-rich group that embraces representatives of, e.g. the Isopoda, Amphipoda and Mysida (“opossum shrimps”), taxa whose neuroanatomy has not been carefully examined. The current study sets out to analyze brain morphology of the mysid *Neomysis integer* (Leach, 1814; Peracarida, Mysida) using immunohistochemistry against the presynaptic protein synapsin and the neuropeptides RFamide, SIFamide and allatostatin combined with three-dimensional reconstruction of elements of the central olfactory pathway. Furthermore, we studied the inventory of sensilla on the first pair of antennae using cuticular autofluorescence. Anterograde filling with neuronal tracers allowed visualisation the central projections of the sensilla on the first pair of antennae. This species is known to display a sexual dimorphism in both the peripheral and central olfactory pathway. We focussed our analysis on this aspect because in contrast to Hexapoda, reports on a sexual dimorphism of the olfactory system are extremely rare in malacostracan crustaceans. We provide a detailed description of the sensilla associated with a male-specific structure on the pair of first antenna the “*lobus masculinus*”. Furthermore, we analyzed the projection patterns of theses sensilla into the “male-specific neuropil” in the deutocerebrum and critically discuss our results in comparison to examples of sexual dimorphism in the chemosensory pathways in other malacostracan crustaceans and hexapods.

## Introduction

**Fig. 1:**
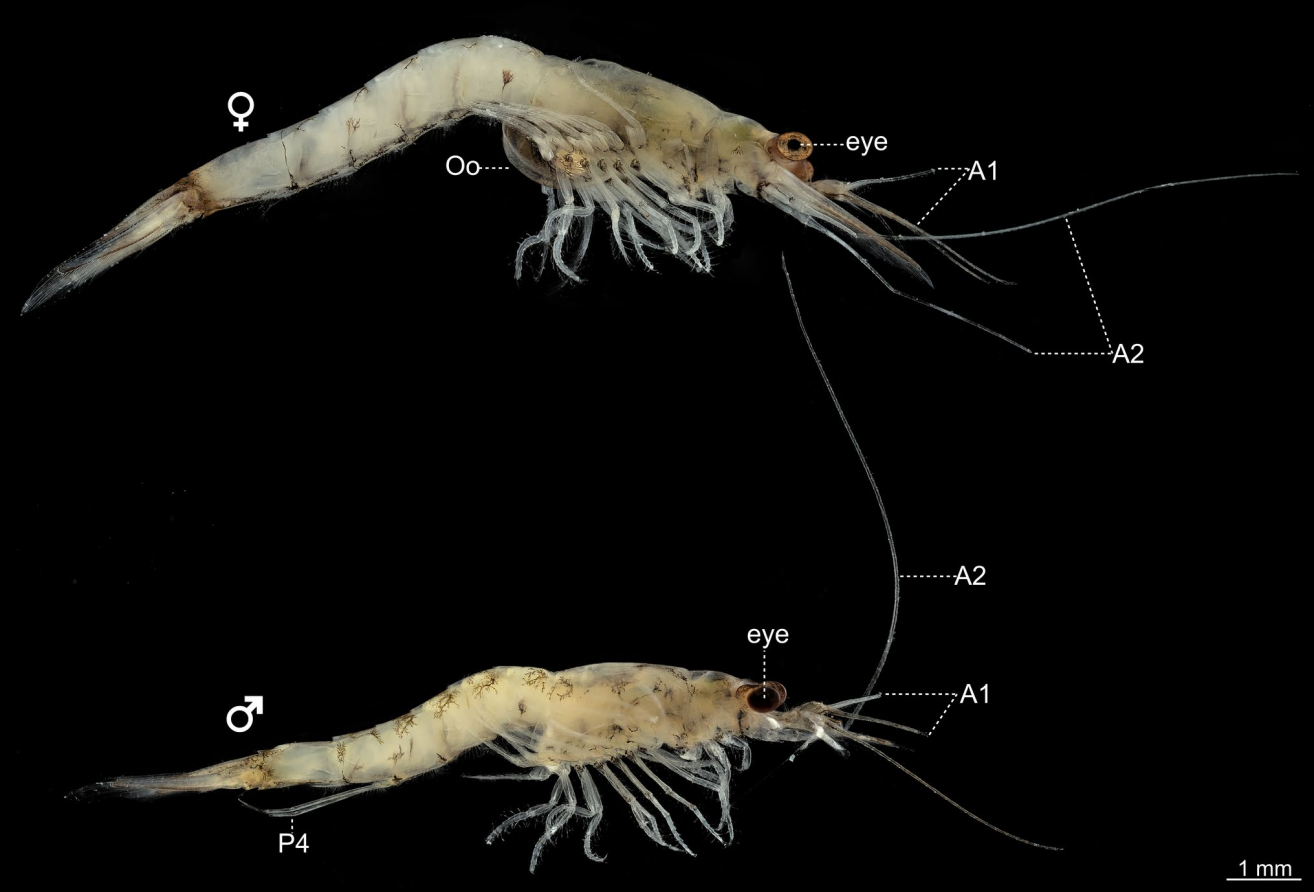
Female and male *Neomysis integer*, lateral view (photograph by Peter Michalik, Greifswald). A1, first antenna; A2, second antenna; Oo, oostegite; P4, fourth pleopod pair

*Neomysis integer* is a member of the Mysida (“opossum shrimps”; Porter 2016) which together with the Amphipoda, Isopoda and other taxa constitute the Peracarida (Wirkner and Richter 2010; Meland et al. 2015; Oliveira et al. 2023). It is one of the most prevalent species in the Wadden Sea and in shallow coastal waters across Europe (Teufert 1980; Mees et al. 1994; Verslycke et al. 2003; Porter et al. 2008; Remerie et al. 2009) and has an s-shaped, transparent, brownish-greenish body (≤ 30 mm), stalked eyes, two pairs of antennae and a lanceolate-shaped telson (Fig. 1; Teufert 1980; Klausnitzer 2019). The animals live in large shoals and mate exclusively at night (Clutter 1969). They are important models in aquatic ecophysiology because they are known to respond to fluctuations of external abiotic and biotic factors with alterations in body size and survival rate (reviewed in Oliveira et al. 2023), testosterone production (Poelmans et al. 2006), embryogenesis (Ghekiere et al. 2007) and ingestion and respiration rate (Hennigs et al. 2022; Brinkop et al. 2024).

Why study the brain of Mysida? Browsing across recent book volumes and reviews on the neurobiology of Crustacea shows a strong bias of our knowledge in this field on representatives of the Decapoda (“ten legged” crustaceans) such as crayfish, crabs, clawed lobsters and spiny lobsters (Breithaupt and Thiel 2010; Strausfeld 2012; Harzsch and Krieger 2018). This bias is particularly prevalent regarding aspects of crustacean sensory systems (reviews Hallberg and Skog 2010; Mellon 2007, 2012, 2014) and their brain morphology (reviews Sandeman et al. 2014; Schmidt 2016). Malacostracan crustaceans inhabit a chemically rich and diverse environment. Their chemical senses are thought to play an essential role in finding food, reproduction and avoiding predators so that research on crustacean olfactory systems is an active discipline which, again, is strongly biased towards studies focussing on decapod crustaceans (reviews, e. g., Schmidt 2007; Schmidt and Mellon 2010; Derby and Weissburg 2014; Derby et al. 2016; Harzsch and Krieger 2018; Derby and Caprio 2024). Therefore, to explore the structural diversity of the chemical senses in malacostracan crustaceans and to provide insight into evolutionary transformations of crustacean chemosensory systems, we recently concerned ourselves with a series of studies focussing on these topics in members of the Peracarida, specifically the Isopoda (Harzsch et al. 2011; Kenning and Harzsch 2013) and Amphipoda (Wittfoth et al. 2019; Raspe et al. 2023; Kümmerlen et al. 2023). Our knowledge of nervous system anatomy in representatives of Mysida—as another major peracarid taxon—is limited. A basic description of the brain of *N. integer* was provided by Aramant and Elofsson (1976). Studies by Johansson and Hallberg (1992a, b) and Johansson et al. (1996) focussed on the anatomy of the peripheral and central olfactory pathway of mysid representatives *Neomysis integer*, *Praunus flexuosus*, *Boreomysis arctica* and *Boreomysis megalops* and provided the first evidence of sexual dimorphism in their chemosensory systems. These authors’ studies showed that *N. integer* possesses male-specific slender sensilla and long hair-like sensilla on a specialized structure of the first antennae, the *lobus masculinus*. What is more, these authors described a male-specific neuropil in the deutocerebrum, situated in close proximity to the olfactory lobe and the lateral antenna 1 neuropil (Johansson and Hallberg 1992a, 1992b). Further reports of sexual dimorphism in crustacean chemosensory systems are scarce, with examples including longer first antennae in males of the isopod *Asellus aquaticus* (Bertin and Cezilly 2003) and in males of the amphipod *Monoporeia affinis* (Hallberg et al. 1997) and longer first antennae and male-specific sensilla in males of the amphipod *Hyperia galba* (Dittrich 1988; Hallberg et al. 1997). In conclusion, members of the Mysida so far provide the only crustacean example displaying a pronounced sexual dimorphism in the peripheral olfactory pathway, specifically the first antenna, as well as an additional neuropil adjacent to the olfactory lobe and the lateral antenna1 neuropil in the brain (Johansson and Hallberg 1992a, 1992b). 

The present study sets out to describe the brain morphology of male and female individuals of *N. integer* in more detail than currently available by using immunohistochemistry against presynaptic proteins and the neuropeptides RFamide, allatostatin and SIFamide combined with confocal laser-scan microscopy, 3D reconstruction and detection of cuticular autofluorescence. We are particularly interested in details of the peripheral and central olfactory pathway to deepen our understanding of sexual dimorphism in crustacean chemosensory systems.

## Material and methods

### Experimental animals

Adult mysids were collected using hand-held nets in the “Dänische Wiek”, a tributary of the Greifswalder Bodden (Germany, Baltic Sea), at a water depth of less than 1 m and immediately stored in an aerated water tank at 4 °C. Identifying the different species is possible by morphological features of their telson as an important determining character (Teufert 1980). *N. integer* was identified by its unique posterior lanceolate pointed-shaped tail (Klausnitzer 2019). Both adult female and male animals were collected, with male *N. integer* recognizable by an extended, single jointed and elongated fourth pleopod pair, whereas the last two or three pairs of legs give rise to the oostegites or brood plates in females (Teufert 1980).

### Immunohistochemistry

Animals were beheaded between the third and fourth thoracic segments and first antennae were removed from the head. For immunohistochemical preparation, the heads were chemically fixed in 4% paraformaldehyde (PFA, Electron Microscopy Sciences, lot 220,923–50) in 0.1 M phosphate-buffered saline solution (PBS) for 48 h at 4 °C. Afterwards, the samples were washed 3 × 10 min in PBS, then infiltrated for 20 min with 1:1 cryprotectant:PBS (500 ml 0.1 M phosphate buffer, 300 g sucrose, 10 g of polyvinyl-pyrrolidone (PVP-40 Sigma), 300 ml of ethylene glycol (enzyme grade Fisher BP-230), and Aqua dest. to reach 1000 ml). Samples were stored at − 20 °C in cryoprotectant until dissection. After careful dissection of the brains, the tissues were washed and blocked with PBS-Tx (PBS with 0.3% TritonX-100; PBS-Tx; Sigma-Aldrich, X100 500 mL, lot STBJ5677, 1% bovine serum albumin; BSA; Sigma, A5253-250G, lot SLBL7392 V) for 2 × 15 min, 2 × 30 min and 1 × 60 min at room temperature (RT) with gentle agitation. Tissues were incubated in primary antiserum in the following antisera mixtures: anti-synapsin SYRNOF1, raised in mouse (1:10 in PBS-Tx) with either anti-A-type Dip allatostatin I (1:2000 in PBS-Tx) raised in rabbit or anti-RFamide (1:2000 in PBS-Tx) or anti-SIFamide (1:750 in PBS-Tx) raised in rabbit. Tissues were incubated in antisera for 3.5 days in the dark at RT with gentle agitation. All samples were washed with PBS-Tx with BSA for 2 × 10 min and 2 × 20 min at RT. Subsequently, tissues were incubated in secondary antisera for 2.5 days (goat anti-rabbit Alexa Fluor™ 488, Invitrogen; goat anti-mouse Cy3, Jackson ImmunoResearch; each diluted 1:500 in PBS-Tx). Nuclei were counterstained using Hoechst 33,342 (Thermo Fisher Scientific, cat# 62,249, diluted 1:10,000). After incubation, the brains were washed with PBS-Tx including 1% bovine serum albumin (BSA) for 2 × 10 min, 2 × 20 min and an overnight washing step. For embedding, all samples were infiltrated with 1:1 PBS:glycerol for 60 min, followed by 20 min of 1:9 PBS:glycerol solution. Tissues were embedded in DABCO-glycerol (DABCO, for 1000 µl: 200 µl Aqua dest., 50 mg DABCO, 800 µl glycerol; Carl Roth, lot 294,216,515). As a control, the first antibody was replaced by PBS-Tx with BSA, resulting in all labeling being abolished.

First antennae were removed from the head capsule, washed and incubated in PBS-Tx 1% BSA for 2 × 15 min, 2 × 30 min and 1 × 60 min at RT with gentle agitation. Nuclei were counterstained using SYTOXTM Green (dilution 1:20,000 in PBS-Tx, InvitrogenTM, Thermo Fisher Scientific #S7020). Antennae were washed, infiltrated with and then embedded in DABCO-glycerol.

### Specificity of the antisera

#### Synapsin

The monoclonal anti-*Drosophila* synapsin SYRNOF1 antibody (Developmental Hybridoma Bank (DSHB) Hybridoma Product 3 C11, anti-SYRNOF1 as deposited to the DSHB by E. Buchner, University Hospital Würzburg, Germany; supernatant) was raised against *Drosophila melanogaster* GST-synapsin fusion protein in mouse. Western blot analysis of *Drosophila melanogaster* head homogenates showed that the SYRNOF1 antibody recognizes four different synapsin isoforms (70, 74, 80 and 143 kDa) (Klagges et al. 1996). Other studies revealed that the epitope which is recognized by “SYNORF1” is conserved within arthropods (Sullivan et al. 2007; Harzsch and Hansson 2008). Immunohistochemical studies using the SYNORF1 antiserum by Kümmerlen et al. (2023), Krieger et al. (2010) and Polanska et al. (2020) demonstrate that Malacostraca exhibit similar patterns in nervous tissue structures. However, no additional experiments were performed to verify the specificity of this antiserum in *N. integer*. Consequently, we refer to the observed labeling as synapsin-like immunoreactivity (synapsin-like IR).

#### Allatostatin A-like peptides

In insects and crustaceans, the A-type allatostatins comprise a diverse family of neuropeptides characterized by the C-terminal motif -YXFGLamide (Nässel and Homberg 2006; Stay and Tobe 2007). We used antibodies raised against A-type Dipallatostatin I, ASPSGAQRLYGFGLamide conjugated to bovine thyroglobulin using glutaraldehyde (Vitzthum et al. 1996). Non-competitive ELISAs had confirmed its specificity by showing no cross-reactivity with CCAP, FMRFamide, leucomyosuppression, locustatachykinin 11, perisulfakinin or proctolin. As we did not follow up with experiments for the specificity of the antibody in *N. integer*, we refer to the observed labeling as “allatostatin-like IR”.

#### FMRFamide-like peptides

Peptides of the FMRFamide family are found in both invertebrates and vertebrate representatives (Greenberg and Price 1979). More than 50 members of the family share a common characteristic amino acid pattern: a carboxy-terminal arginine (R) and an amidated phenylalanine (F) motif (reviewed in Greenberg and Price 1992; Nässel 1993; Dockray 2004; Nässel and Homberg 2006). The polyclonal antibody used in this study was raised in rabbit against synthetic FMRFamide conjugated to bovine thyroglobulin. According to the manufacturer and experiments with the decapod crustacean *Coenobita clypeatus* (Harzsch and Hansson 2008), preadsorption of the diluted antibody with 100 µg/ml of FMRFamide completely abolishes all immunohistochemical labeling. Given that the carboxyterminal sequence RFamide is a common feature among crustacean FMRF-related peptides, we anticipate the antiserum to detect peptides terminating with the sequence RFamide. As we did not follow up with experiments for the specificity of the antibody in *N. integer*, we designate the labeled structures in our specimens as “RFamide-like IR”.

#### SIFamide-like peptides

The myotropic neuropeptide SIFamide (AYRKPPFNGSLF-amide sequence) was first discovered in the grey flesh fly (Janssen et al. 1996). A homologous SIFamide sequence (GYRKPPFNGSIFamide) was found in malacostracan crustaceans such as *Procambarus clarkii* (Yasuda et al. 2004). Although the specificity of the antibody has not been confirmed, the staining pattern of the antibody overlaps with MALDI-TOF MS analysis of the stained tissue (Yasuda et al. 2004; Yasuda‐Kamatani and Yasuda 2004). The antibodies against the antigen (Cys)GYRKPPFNGSIF-CONH2 were conjugated to BSA as a carrier with a rabbit as host (Yasuda et al. 2004; Polanska et al. 2007). Preadsorption experiments with SIFamide in the peracarid crustacean *Parhyale hawaiensis* led to a complete extinction of the staining (Raspe et al. 2023). As we did not follow up with experiments for the specificity of the antibody in *N. integer*, we will refer to the observed labeling as “SIFamide-like IR”.

### Anterograde fills

Mass anterograde filling of the first antennae was carried out after fixing the heads of *N. integer* in 2% PFA in PBS with 5% glucose for 1 to 5 days. Either the medial or lateral antenna was cut above the peduncle, and DiI crystals (1,1′-Dioctadecyl-3,3,3′,3′-tetramethylindocarbocyaninperchlorat, Sigma 42,364-100MG) were manually placed on the cut side of the antenna proximal to the brain. The preparations were incubated in PBS at 4 °C for 21–22 days to allow sufficient time for the dye to travel along the axonal cell membranes into the brain. Subsequently, brains were dissected, washed in PBS at least 3 × 10 min, mounted in DABCO-glycerol and scanned with an excitation wavelength of 561 nm.

### Imaging and 3D reconstruction

All samples were scanned with a Leica TCS SP5 II confocal laser scanning microscope equipped with a DPSS-, 405 Diode- and argon laser. The wavelengths of laser lines were adjusted to 405 nm to scan Hoechst 33,342 as well as cuticular autoflourescence, to 561 nm for scanning Cyanine3 and to 488 nm for Alexa Fluor^TM^488. The Leica Application Suite Advanced Fluorescence was applied as software package to drive the microscope (LAS AF 2.7.3). The offset, gain, zoom and laser intensity were individually set depending on the preparation and laser line to obtain an optimal image. Photon loss deeper in the tissue was compensated with a linear compensation function increasing the laser intensity depending on the tissue thickness. Overview scans of the whole brain were scanned with 20x magnification and a resolution of 1024 × 1024 pixels, resulting in a pixel size of 0.76 µm and a *z*-depth of 0.63 µm. Details were scanned with a resolution of 2024 × 2024 pixels, resulting in a pixel size for the × 40 magnification of 0.19 µm and a *z*-depth of 0.30 µm and for the 63x magnification of 0.085 µm and a *z*-depth of 0.30 µm. At least five successfully stained samples per sex and antibody combination were scanned and analyzed. Image projections were globally enhanced for contrast and brightness with the software ImageJ (ImageJwin64, Version 2.9.0 by Fiji, Schindelin et al. 2012) and used for analysis.

For the whole brain reconstruction as well as to quantify the number and shape of the olfactory glomeruli, image stacks of the brain were manually segmented in Amira (Version 6.0.1, FEI Visualization Science Group, Thermo Fisher Scientific). Labeling was performed on every fifth plane, followed by interpolation. The selected volume was then assigned to a material. For the presentation of the olfactory glomeruli, the surface was smoothened and calculated.

### Nomenclature

The nomenclature for all neuroanatomical structures of the brain of *N. integer* is based on Meth et al. (2017), Kenning et al. (2013), Sandeman et al. (1992) and Johansson and Hallberg (1992a, 1992b). The nomenclature of the antennae and sensillae follows Hallberg et al. (1997). Cell clusters in all images were named according to Kenning and Harzsch (2013). The terms lamina, medulla and lobula have been used for the visual neuropils instead of lamina ganglionaris, medulla externa and medulla interna according to Harzsch (2002). Additionally, we used the term “lateral protocerebrum” instead of “hemiellipsoid body” in reference to Harzsch and Krieger (2021). The body axis was used to describe the orientation of the brain. In *N. integer*, the body axis is separated from the neuroaxis (Ito et al. 2014). To facilitate orientation and enhance clarity, images of paired structures were mirrored to show the right side.

## Results

### Gross morphology of the brain

**Fig. 2:**
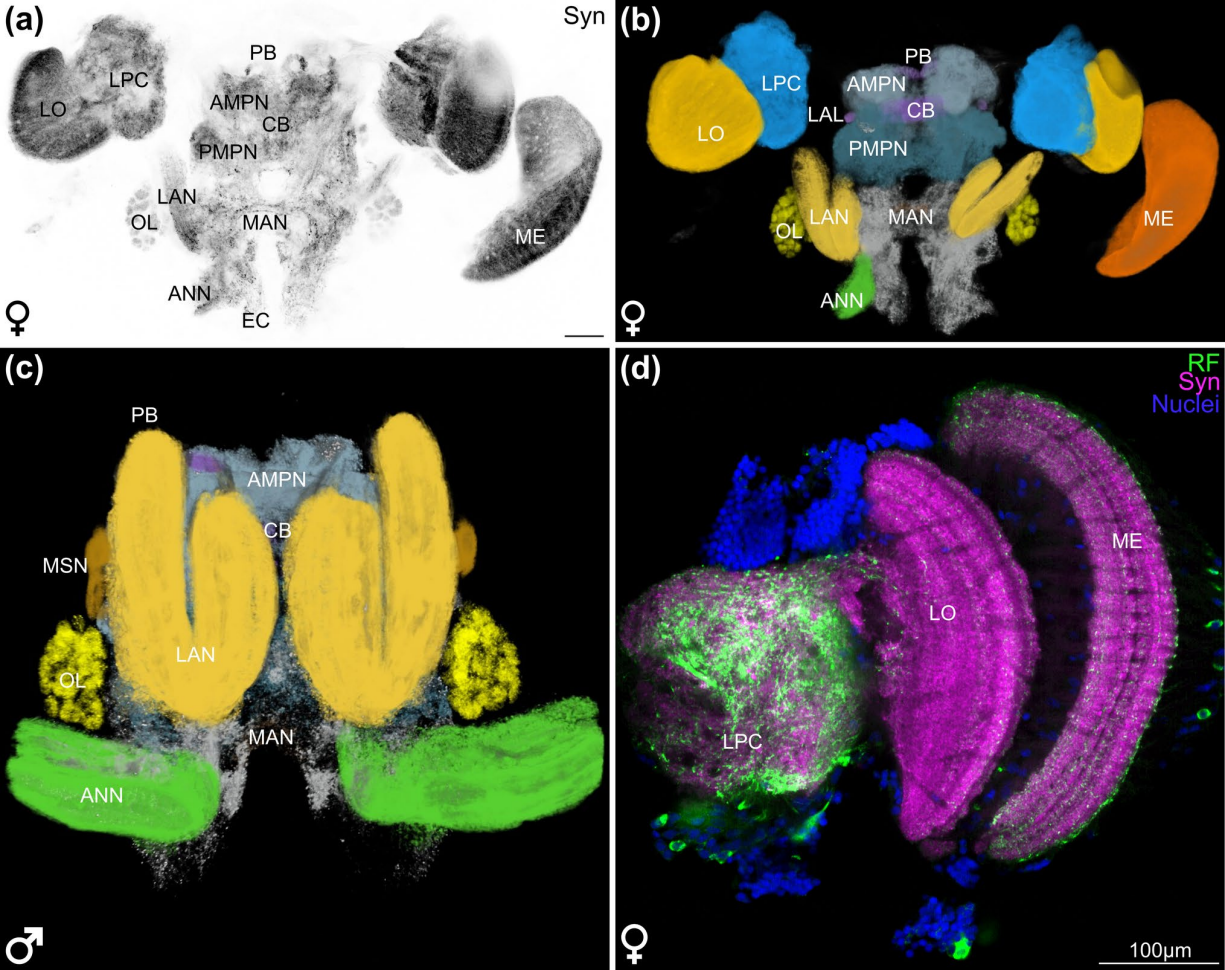
Overview of the brain of *N. integer*. **a** Single optical section (1.3 µm) of a medial plane of a female brain, black and white-inverted image showing synapsin-like immunoreactivity. **b** AMIRA reconstruction of the synapsin-like immunoreactivity of a female’s brain, false colours highlight neuropils. **c** AMIRA reconstruction of the synapsin-like immunoreactivity of a male’s brain, false colours highlight neuropils. **d** Single optical section (0.63 µm) of the eyestalk neuropils, RFamide-like immunoreactivity in green, synapsin-like immunoreactivity in magenta, nuclei in blue. All brains oriented with dorsal towards the top, all scale bars 100 µm. AMPN, anterior medial protocerebral neuropil; ANN, antenna 2 neuropil; CB, central body; EC, esophageal connective; LAL, lateral accessory lobe; LAN, lateral antenna 1 neuropil; LO, lobula; LPC, lateral protocerebrum; MAN, medial antennae 1 neuropil; ME, medulla; MSN, male-specific neuropil; OL, olfactory lobe; PB, protocerebral bridge; PMPN, posterior medial protocerebral neuropil; RF, RFamide-like immunoreactivity; Syn, synapsin-like immunoreactivity

The head of *Neomysis integer* is equipped with bilaterally paired, large antennae in both sexes (Fig. 1). The paired first antennae (A1) are composed of two flagella. The uniramous paired second antennae (A2) are about twice as long as the first antennae. The head is also equipped with bilaterally paired and mobile eyestalks that bear the compound eyes (Fig. 1). The brain of *N. integer* consists of two spatially separated parts. The neuropils of the lateral protocerebrum and the visual neuropils lamina (which unfortunately was often lost during preparation), lobula and medulla are located in the eyestalks (Fig. 2a, b and d). The neuropils in the eyestalks are connected to the second part, the median brain (Fig. 2c), by the protocerebral tract that contains neurites, some of which display allatostatin-like immunoreactivity (Fig. 8d).

**Fig. 3:**
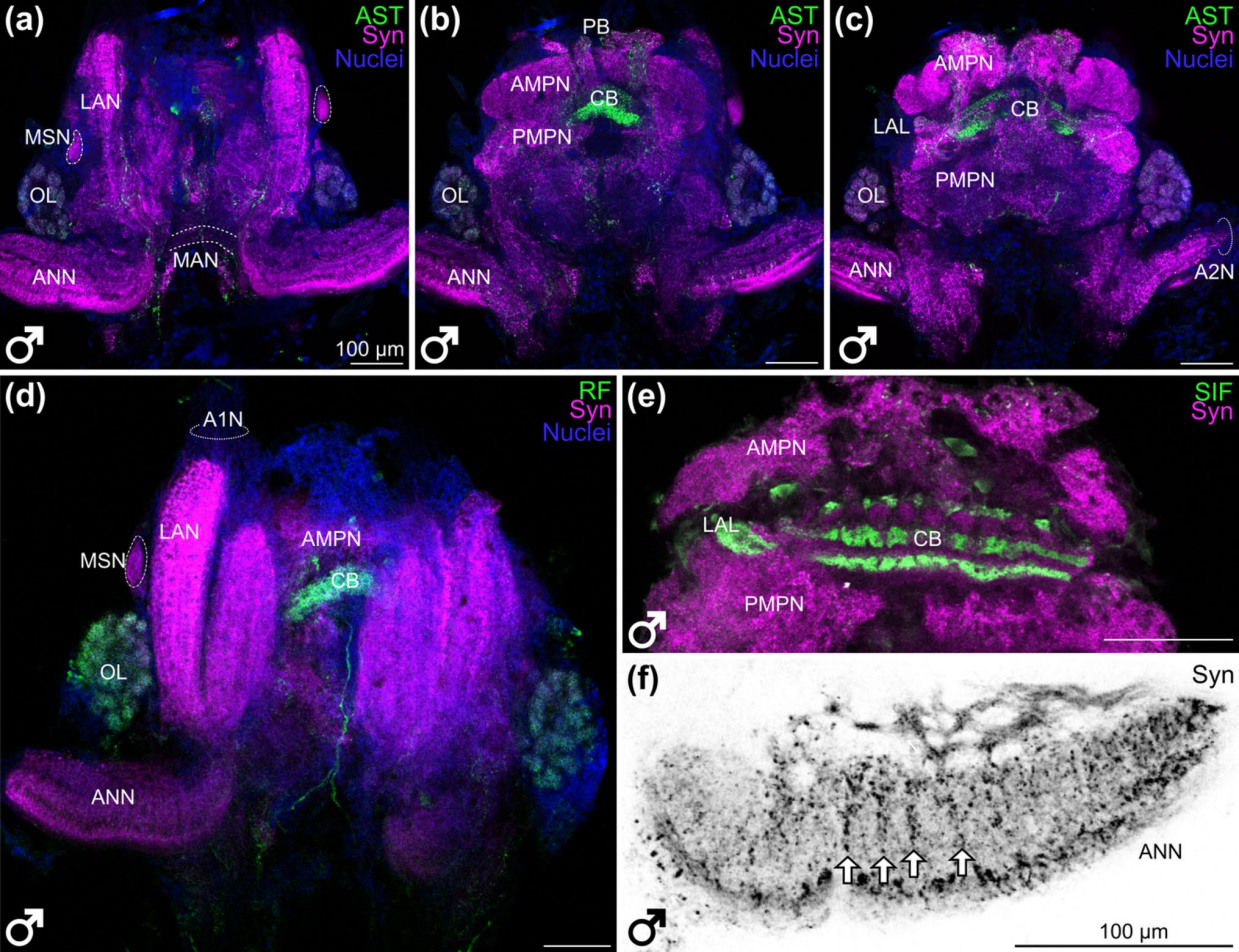
Overview of the brain of *N. integer*. **a** Single optical section (0.63 µm) of an anterior vibratome section of a male brain; green, allatostatin-like immunoreactivity; magenta, synapsin-like immunoreactivity; blue, nuclei. **b** Single optical section (0.63 µm) of a medial plane of male’s brain, green, allatostatin-like immunoreactivity, magenta, synapsin-like immunoreactivity, blue, nuclei. **c** Single optical section (0.63 µm) of a posterior plane of male brain, green, allatostatin-like immunoreactivity, magenta, synapsin-like immunoreactivity, blue, nuclei. **d** Single optical section (0.63 µm) of an anterior plane of male’s brain, green, RF-like immunoreactivity, magenta, synapsin-like immunoreactivity, blue, nuclei. **e** Single optical section (0.63 µm) of a posterior plane of a brain, green, SIFamide-like immunoreactivity, magenta, synapsin-like immunoreactivity. **f** Single optical section (0.63 µm) of the synapsin-like immunoreactivity in the antenna 2 neuropil, arrows highlight striated pattern. All brains oriented with dorsal towards the top, all scale bars 100 µm. A2N, antenna 2 nerve; AMPN, anterior medial protocerebral neuropil; ANN, antenna 2 neuropil; AST, allatostatin-like immunoreactivity; CB, central body; LAL, lateral accessory lobe; LAN, lateral antenna 1 neuropil; MAN, medial antennae 1 neuropil; MSN, male-specific neuropil; OL, olfactory lobe; PB, protocerebral bridge; PMPN, posterior medial protocerebral neuropil; RF, RFamide-like immunoreactivity; SIF, SIFamide-like immunoreactivity; Syn, synapsin-like immunoreactivity

**Fig. 4:**
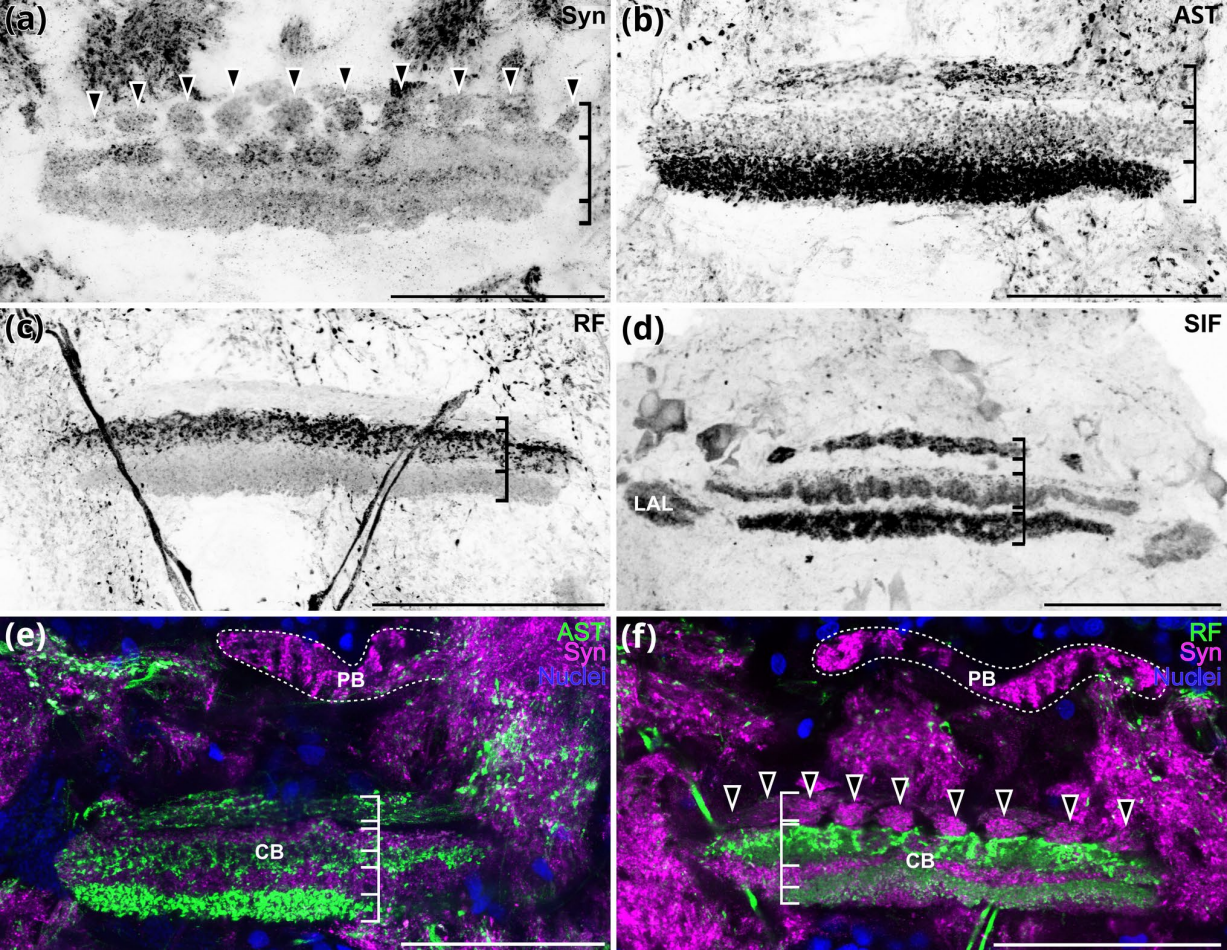
Details of brain morphology of *N. integer*. **a** Maximum projection (2.4 µm) of the synapsin-like immunoreactivity of the central body, layered structure is highlighted with lines, black arrowheads highlight ten distinct substructures in the dorsal-most layer of the central body. **b** Maximum projection (17.7 µm) of allatostatin-like immunoreactivity of the central body, layers indicted with lines. **c** Maximum-projection (21.6 µm) of RFamide-like immunoreactivity in the central body, layers indicated with lines. **d** Maximum projection (30.3 µm) of the SIFamide-like immunoreactivity of the central body and the lateral accessory lobe, layers indicated by line. **e** Maximum projection (2.7 µm) of an overlay of the allatostatin-like immunoreactivity, synapsin-like immunoreactivity and nuclear counterstain of the central body and the protocerebral bridge (dashed line), white lines indicate horizontal layers. **f** Single optical section (0.3 µm) of an overlay of the RFamide-like immunoreactivity, synapsin-like immunoreactivity and nuclear counterstain of the central body and the protocerebral bridge (dashed line), white lines indicate layers, arrowheads indicate round structures in the dorsal-most layer of the central body. All brains oriented with dorsal towards the top, all scale bars 100 µm. AST, allatostatin-like immunoreactivity; ANN, antenna 2 neuropil; CB, central body; LAL, lateral accessory lobe; PB, protocerebral bridge; RF, RFamide-like immunoreactivity; SIF, SIFamide-like immunoreactivity; Syn, synapsin-like immunoreactivity

The median protocerebrum is composed of the anterior medial protocerebral neuropil (AMPN) and the posterior medial protocerebral neuropil (PMPN) that circumscribe the central body (CB), a medial unpaired neuropil. Likewise, the bilaterally paired lateral accessory lobes (LAL) and, located dorsomedially, the protocerebral bridge (PB) are part of the median protocerebrum (Figs. 2a–c and 3a–e). The PB has a striated appearance when labelled for synapsin-like immunoreactivity (Fig. 4e, f). The lateral accessory lobes display strong SIFamide-like immunoreactivity (Figs. 3e and 4d). The AMPN, clearly demarcated by synapsin-like immunoreactivity (Fig. 3c, e), has very little immunoreactivity for the neuropeptides allatostatin (3c), RFamide (Fig. 3d) and SIFamide (Fig. 3e). The CB is located medial to the boundary between the AMPN and PMPN (Figs. 2a–c and 3b–e). Anteriorly, it consists of about ten transversely arranged subunits, as visualized by synapsin-like immunoreactivity (Fig. 4a and f, arrowheads). Labelling against the neuropeptides allatostatin (Fig. 4b), RFamide (Fig. 4c) and SIFamide (Fig. 4d) revealed three distinct transverse layers in the CB. However, these three layers display different relative intensities for each neuropeptide (Figs. 4b–f). Allatostatin-like immunoreactivity is most pronounced in the ventral layer (Fig. 4b), RFamide-like immunoreactivity is most pronounced in the medial layer (Fig. 4c) and SIFamide-like immunoreactivity is pronounced in all three layers (Fig. 4d). When the signal of the neuropeptides is overlaid with the channel showing synapsin-like immunoreactivity, the three layers do not overlap: the central body appears to have at least five layers in the allatostatin-like and synapsin-like overlay (Fig. 4e), at least four layers in the RFamide-like and synapsin-like immunoreactivity (Fig. 4f) and at least four layers in the SIFamide-like and synapsin-like immunoreactivity overlay (Fig. 3e).

The deutocerebrum of *N. integer* is dominated by the bilaterally paired, elongated and medially split lateral antenna 1 neuropils (LAN) (Figs. 2a–c and 3a, d) which receive input *via* the antenna 1 nerve. Next to the LAN and slightly more posteriorly, the bilaterally paired olfactory lobes (OL) are located (Figs. 2a–c and 3a–d) which are structured in spherical subunits, the olfactory glomeruli. Next to the LAN and dorsal to the OL, additional bilaterally paired neuropils are apparent in male *N. integer*, the male-specific neuropil (MSN, Fig. 3a, d; see below for more details). Ventromedially to the LAN, the unpaired medial antenna 1 neuropil (MAN) is located (Figs. 2a–c and 3a). The tritocerebrum is dominated by the elongated, bilaterally paired antenna 2 neuropils (ANN; Figs. 2c and 3a–f). The ANN is located ventrally to the deutocerebrum, receives input from the antenna 2 nerve, and displays a striated pattern of synapsin-like immunoreactivity (Fig. 3f).

### Sensilla on the first pair of antennae display a sexual dimorphism

**Fig. 5:**
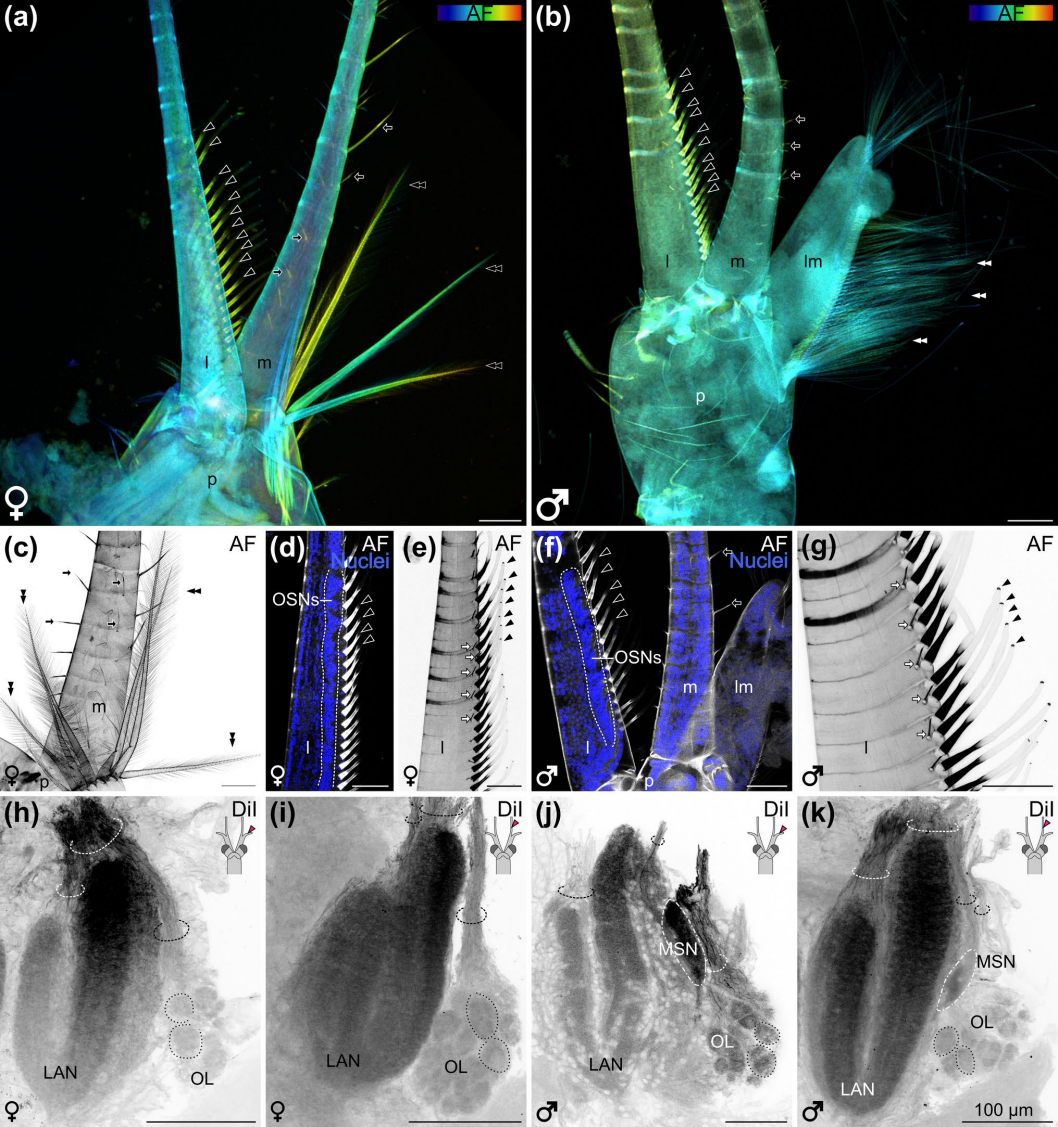
First antenna and anterograde fill. **a** Depth-coded maximum projection (covering 104 µm) of the autofluorescence of the first antenna of a female, with the split medial and lateral flagellum. **b** Depth-coded maximum projection (covering 41.6 µm) of the autofluorescence of the first antennae of a male, with the split medial and lateral flagellum. **c** Maximum projection (covering 39.69 µm) of the autofluorescence of the peduncle, the medial antenna and the female-specific sensilla (black double arrowhead) on the peduncle of the first antennae of a female. **d** Overlay of a single optical section (0.3 µm) of the autofluorescence (grey) and the nuclei (blue) in the medial antenna of a female. OSNs highlighted with dotted line, aesthetascs highlighted with black arrowheads. **e** Maximum projection (covering 12.6 µm) of the autofluorescence of the medial flagellum of a female, aesthetascs highlighted with black arrowheads, slender sensilla highlighted with white arrows. **f** Overlay of a single optical section (0.63 µm) of autofluorescence (grey) and nuclei (blue) of the peduncle and the proximal region of the medial and lateral flagellum of a male. OSNs highlighted with dotted line, aesthetascs highlighted with black arrowheads. **g** Maximum projection (covering 28.2 µm) of the autofluorescence of the medial flagellum of a male. Slender sensilla highlighted with white arrows, aesthetascs highlighted with black arrowheads. **h** Maximum projection (covering 26.1 µm) of a DiI anterograde fill from the lateral antenna (see insert) of a female. Axon bundles are highlighted with dashed half circles, olfactory glomeruli with dotted line. **i** Maximum projection (36.9 µm) of a DiI anterograde fill from the medial antenna (see insert) of a female. Axon bundles are highlighted with dashed half circles, olfactory glomeruli with dotted line. **j** Maximum projection (7.56 µm) of a DiI anterograde fill from the lateral antenna (see insert) of a male. Axon bundles are highlighted with dashed half circles, olfactory glomeruli with dotted line. **k** Maximum projection (56.1 µm) of a DiI anterograde fill from the medial antenna (see inset) of a male. Axon bundles are highlighted with dashed half circles; olfactory glomeruli with dotted line; black arrowheads with white outline, aesthetascs; black arrow with white outline, simple sensilla; white arrow with black outline, slender sensilla; black double arrowhead with white outline, female-specific sensilla; white double arrowhead with black outline, male specific sensilla. All antennae are oriented with distal towards to the top, proximal towards the bottom, all brains oriented with dorsal towards the top, all scale bars 100 µm. AF, autofluorescence; l, lateral antenna; LAN, lateral antenna 1 neuropil; lm, *lobus masculinus*; m, medial antenna; MSN, male-specific neuropil; OL, olfactory lobe; OSNs, olfactory sensory neurons; p, peduncle

The bilaterally paired first antennae are part of the chemosensory system of *N. integer* and are composed of lateral and medial flagella (Fig. 5a, b). A sexual dimorphism is apparent regarding structures on the peduncle of the first antennae: female individuals have multiple long, plumose sensilla with a “feather-like” appearance (Fig. 5a, c, double arrow heads) whereas males possess an extension on the peduncle of the first antennae, which we call “*lobus masculinus*” in accordance with Johansson and Hallberg (1992a; see Fig. 5b, f). The *lobus masculinus* displays a multitude of thin, long, simple sensilla (Fig. 5b, double arrow heads). On the medial flagellum, multiple long and thin simple sensilla are located, in both female and male individuals (Fig. 5a–c, f, black arrows). These sensilla are smooth without any substructures located on them (see also Garm 2004). The lateral flagellum houses the rod-shaped aesthetascs, specialized sensilla for olfaction (Fig. 5a, b, d–g). The aesthetascs do not display any morphological differences between female and male individuals. The basal third of each aesthetasc displayed a high level of autofluorescence, which is distally adjoined by a region of weak autofluorescence and a more prominently fluorescing tip (Fig. 5a, b, d–g, black arrowheads). Between the aesthetascs on the lateral antenna, thin sensilla are observed in female and male individuals (Fig. 5e, g, white arrows). In accordance with Johansson and Hallberg (1992a), we call these thin sensilla “simple sensilla”. The nuclear counterstain revealed the presence of clustered olfactory sensory neurons associated with the aesthetascs in the lateral antenna (Fig. 5d, f).

### Anterograde fills with a neuronal tracer reveal the antennal input to the brain

To visualize the afferent projections of the first antennae to the brain, mass anterograde fills from the third basal-most segment of either the lateral or medial flagellum were conducted for at least five individuals of both sexes. In female individuals, fills from the lateral and the medial antenna resulted in staining of both lobes of the split LAN and of the OL (Fig. 5h, i). Three nerve bundles were observed entering the LAN and the OL from an anterior-dorsal direction (Fig. 5h, i, dashed half circles). In fills from either the lateral or the medial flagella, individual olfactory glomeruli were labelled in the olfactory lobe (Fig. 5h, i, dotted circles). In male individuals, staining was observed in both lobes of the LAN, the O and the male-specific neuropil (MSN; Fig. 5j, k, dotted and dash-dotted line). Without any differences between anterograde fills from the lateral or the medial flagella (Fig. 5j, k), two neurite bundles per half-lobe innervate the LAN. Furthermore, one nerve targets the OL, and one nerve the MSN (Fig. 5j, k, dashed half-circles).

### Immunochemistry reveals the neurochemistry of the central chemosensory pathway

**Fig. 6:**
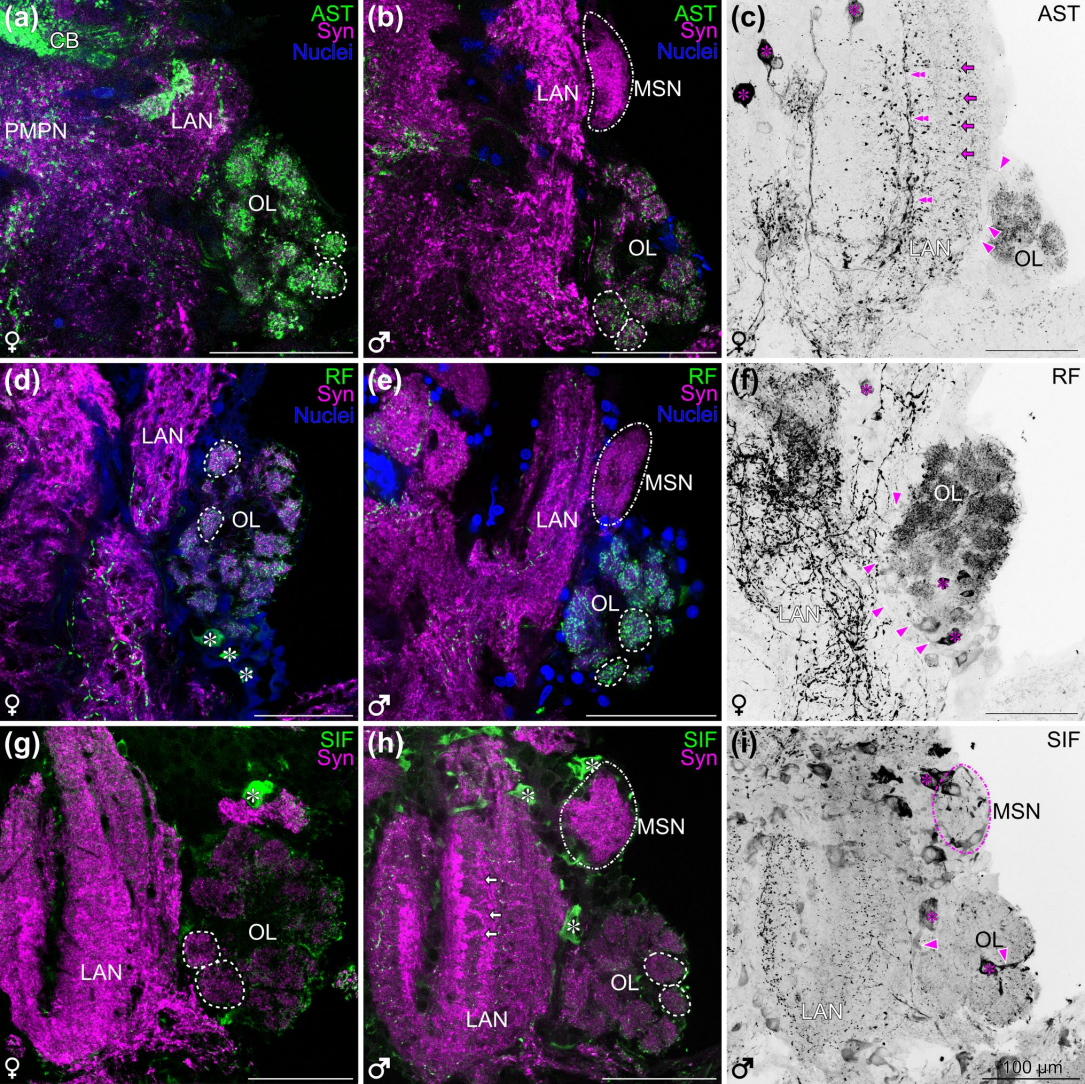
Neuropeptides in the deutocerebrum of *N. integer*. **a** Overlay of a single optical section (0.3 µm) of allatostatin-like immunoreactivity (green), synapsin-like immunoreactivity (magenta) and nuclear dye (blue) of the olfactory lobe, parts of the median protocerebrum and deutocerebrum of a female. **b** Overlay of a single optical section (0.3 µm) of allatostatin-like immunoreactivity (green), synapsin-like immunoreactivity (magenta) and nuclear dye (blue) of the olfactory lobe, parts of the median protocerebrum and deutocerebrum of a male. **c** Maximum projection (covering 34.8 µm) of allatostatin-like immunoreactivity (black), of the olfactory lobe and deutocerebrum of a female. **d** Overlay of a single optical section (0.3 µm) of RFamide-like immunoreactivity (green), synapsin-like immunoreactivity (magenta) and nuclear dye (blue) of the olfactory lobe, parts of the median protocerebrum and deutocerebrum of a female. **e** Overlay of a single optical section (0.3 µm) of RFamide-like immunoreactivity (green), synapsin-like immunoreactivity (magenta) and nuclear dye (blue) of the olfactory lobe, parts of the median protocerebrum and deutocerebrum of a male. **f** Maximum projection (66.9 µm) of allatostatin-like immunoreactivity (black), of the olfactory lobe, parts of the median protocerebrum and deutocerebrum of a female. **g** Overlay of a single optical section (0.3 µm) of SIFamide-like immunoreactivity (green), synapsin-like immunoreactivity (magenta) and nuclear dye (blue) of the olfactory lobe, parts of the median protocerebrum and deutocerebrum of a female. **h** Overlay of a single optical section (0.3 µm) of SIFamide-like immunoreactivity (green), synapsin-like immunoreactivity (magenta) and nuclear dye (blue) of the olfactory lobe, parts of the median protocerebrum and deutocerebrum of a male *N. integer*. **i** Maximum projection (covering 26.1 µm) of SIFamide-like immunoreactivity (black), of the olfactory lobe and deutocerebrum of a male *N. integer*. All brains oriented with dorsal towards the top, all scale bars 100 µm, olfactory glomeruli highlighted with dashed line, male-specific neuropil is highlighted with dash-dotted line, asterisks indicate somata. AST, allatostatin-like immunoreactivity; CB, central body; LAN, lateral antenna 1 neuropil; MSN, male specific neuropil; OL, olfactory lobe; PMPN, posterior medial protocerebral neuropil; RF, RFamide-like immunoreactivity; SIF, SIFamide-like immunoreactivity; Syn, synapsin-like immunoreactivity

Next, we took a closer look at the neurochemistry of the chemosensory neuropils (Fig. 6). In both female and male individuals, strong allatostatin-like immunoreactivity and strong RFamide-like immunoreactivity were observed in the olfactory glomeruli of the olfactory lobe (Fig. 6a–f). Within the glomeruli, allatostatin-like and RFamide-like immunoreactive material was arranged in granular patches (Fig. 6a–f). The signal of RFamide-like immunoreactivity most likely originated from multiple local olfactory interneuron somata located ventrally of the OL that extend neurites that enter the OL (Fig. 6d, f). The somata of allatostatin-like immunoreactive neurons were observed anteromedially to the LAN, with thin neurites extending between the OL and the LAN (Fig. 6c, arrowheads). The LAN showed a slight striation of allatostatin-like immunoreactive material with more prominent neurites extending dorsoventrally along the length of the neuropil (Fig. 6c, double arrowheads and arrows). Synapsin-like immunoreactivity also revealed a transverse striated pattern within the LAN (Fig. 6h) which also displays a network of neurites with strong RFamide-like immunoreactivity (Fig. 6f). Numerous somata with SIFamide-like immunoreactivity surround the LAN and OL in female and male individuals (Fig. 6g–i) but little SIFamide-immunoreactivity was observed in either of these neuropils or the olfactory glomeruli (Figs. 6g–i, glomeruli indicated with dashed lines). The soma of a prominent local olfactory interneuron with SIFamide-like immunoreactivity projects a neurite into that neuropil (Fig. 6i: neuron towards the right, indicated with an asterisk and arrowheads). Another more medially located neuron extends a neurite posteriorly that bypasses the OL (Fig. 6i; neuron towards the left, indicated with an asterisk and arrowheads). Allatostatin-like and RFamide-like immunoreactivity is absent in the MSN (Fig. 6b, e). Even though the MSN is surrounded by somata with SIFamide-like immunoreactivity, little SIFamide immunoreactivity was observed within that neuropil (Fig. 6h, i). For the studied neuropeptides, the staining between female and male individuals was identical in comparable structures.

### Three-dimensional reconstruction and quantification of olfactory glomeruli

**Fig. 7:**
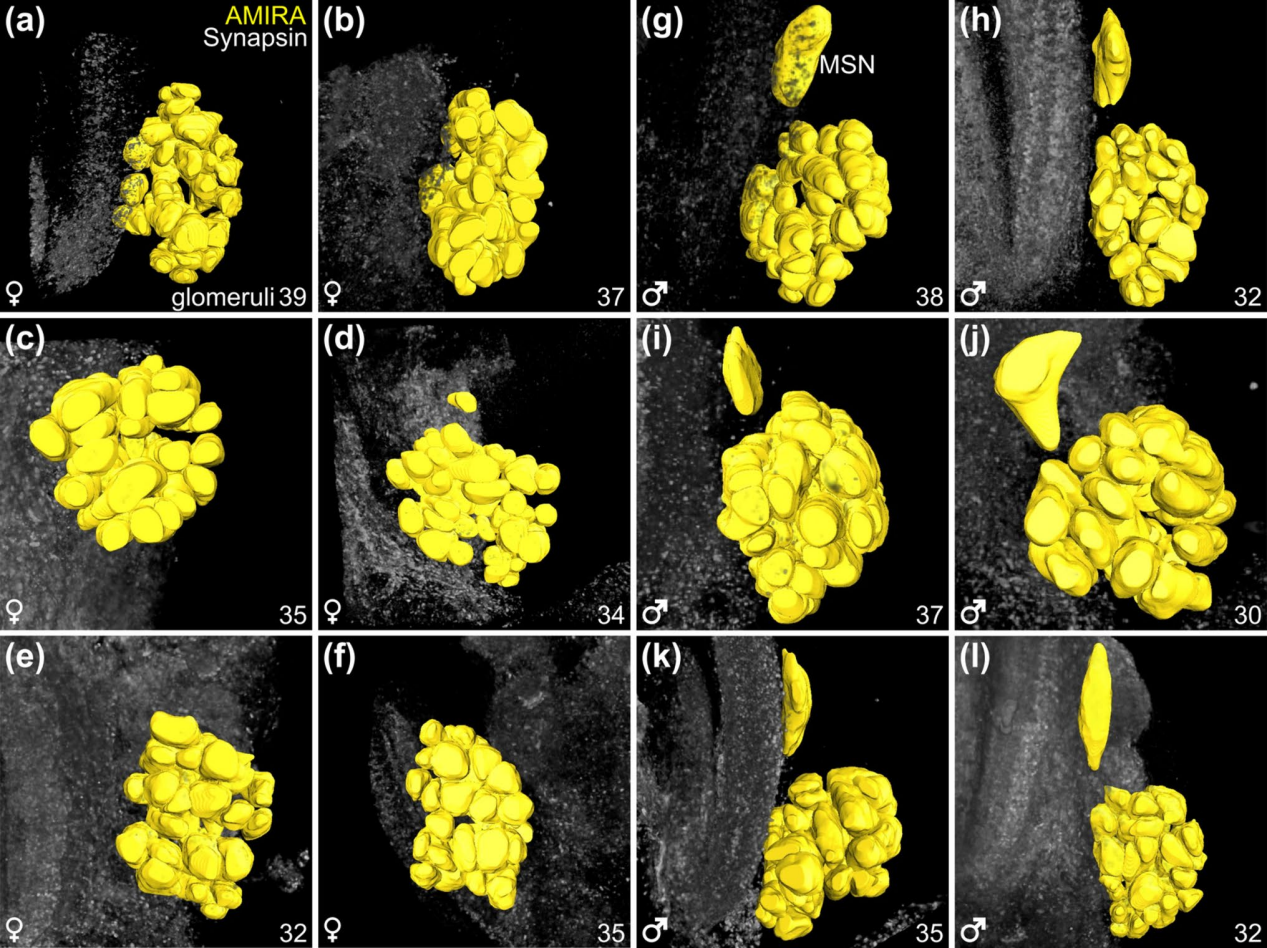
3D reconstruction and quantification of the olfactory glomeruli in six male and six female *N. integer*. Reconstruction of the olfactory glomeruli in yellow and synapsin-like immunoreactivity in grey. **a–f** Female brains. **g–l** Male brains. All brains oriented with dorsal towards the top and anterior towards the front. Number of olfactory glomeruli of the respective olfactory lobe indicated in the bottom right corner. MSN, male-specific neuropil

To contribute insights into the question if olfactory glomeruli are individually identifiable in malacostracan crustaceans and occur in fixed numbers is missing to date (Harzsch and Krieger 2018), we performed three-dimensional reconstructions and quantified their numbers. The reconstruction of individual olfactory glomeruli based on synapsin-like immunoreactivity showed that these structures are roughly spherical in both sexes (Figs. 6 and 7). Furthermore, the synapsin staining revealed a roughly radial arrangement of the olfactory glomeruli around the periphery of the lobe (Fig. 6b, d, g). In all 12 specimens that we reconstructed, it was impossible to individually identify olfactory glomeruli based on their shape or location (Fig. 6). In female individuals, an average of 35.3 ± 2.2 olfactory glomeruli (*N* = 6) per olfactory lobe was counted, and in male individuals, we found an average of 34.0 ± 2.9 olfactory glomeruli (*N* = 6) suggesting no significant difference in the number of olfactory glomeruli between female and male olfactory lobes. However, as mentioned above, male individuals consistently possessed an additional neuropil located posteromedially to the LAN and dorsally to the OL, the MSN (Fig. 6g–i) that was not observed in the brains of any female individuals (Fig. 6a–f). In comparison to male individuals with a male-specific neuropil, no female-specific structure was observed in the brain.

### Neuropils in the lateral protocerebrum

**Fig. 8:**
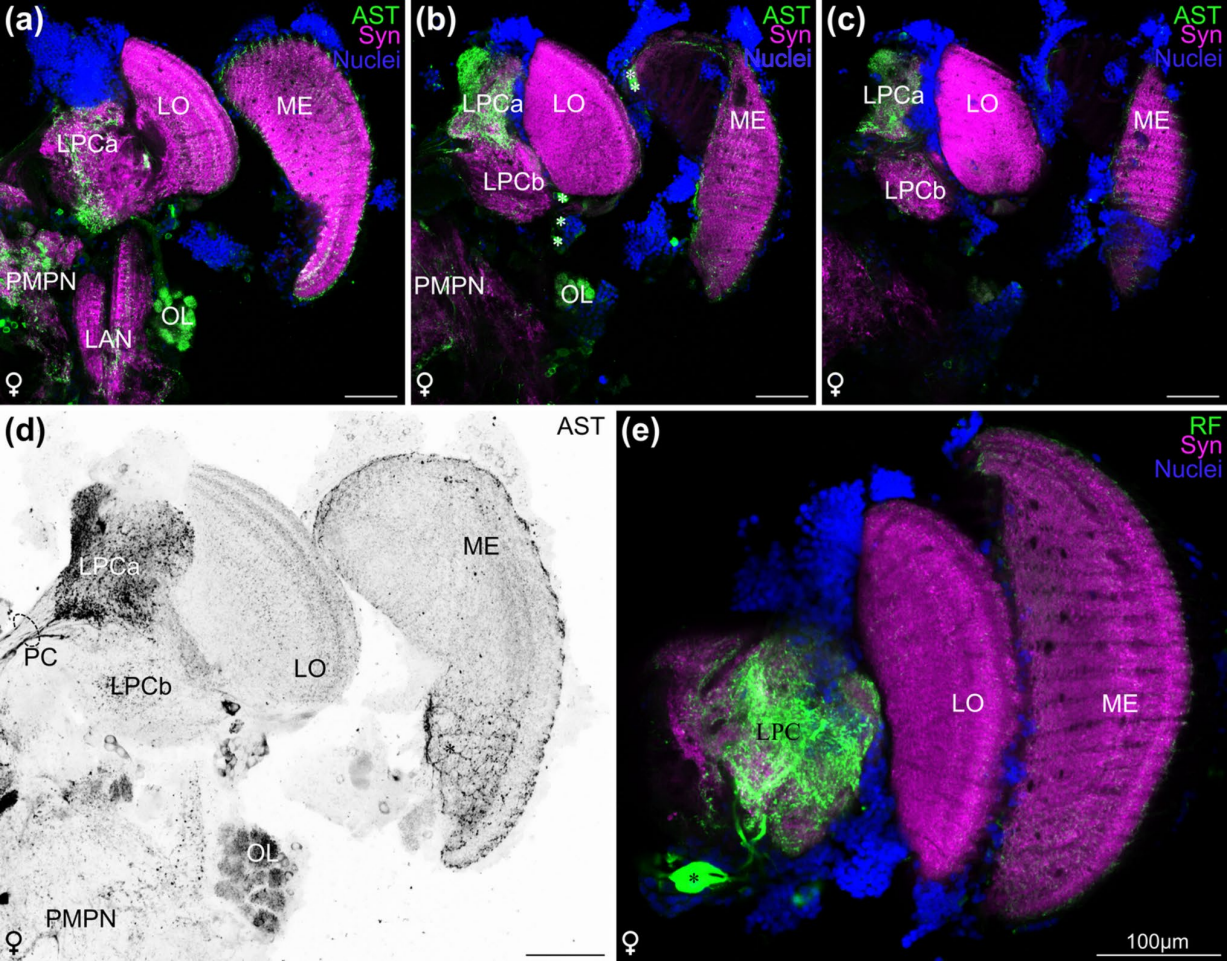
The lateral protocerebrum in *N. integer*. **a–c** Overlay of allatostatin-like immunoreactivity (green, synapsin-like immunoreactivity (magenta) and nuclear dye (blue) of a single optical section (0.63 µm) of an anterior (**a**), medial (**b**) and posterior (**c**) plane of the eye stalk neuropils and part of the median protocerebrum and deutocerebrum. **d** Maximum projection (14.4 µm) of allatostatin-like immunoreactivity (grey), neural tracts highlighted with dashed line. **e** Overlay of RFamide-like immunoreactivity (green, synapsin-like immunoreactivity (magenta) and nuclear dye (blue) of a single optical section (0.63 µm) of the eyestalk neuropils. All brains oriented with dorsal towards the top, all scale bars 100 µm, asterisks indicate somata. AST, allatostatin-like immunoreactivity; CB, central body; LAN, lateral antenna 1 neuropil; LO, lobula; LPC, lateral protocerebrum; LPCa, lateral protocerebrum a; LPCb, lateral protocerebrum b; ME, medulla; MT, medulla terminalis; OL, olfactory lobe; PC, protocerebral connective; PMPN, posterior medial protocerebral neuropil; RF, RFamide-like immunoreactivity; Syn, synapsin-like immunoreactivity

In malacostracan crustaceans, the neuropils of the lateral protocerebrum (LPC), which is located within the eyestalk, are thought to function as higher integrating centres (Sandeman et al. 2014). Based on strong allatostatin-like immunoreactivity in only a part of the LPC, we differentiated this neuropil into two units, the LPCa and LPCb (Fig. 8a–d). Numerous neurites with strong allatostatin-like immunoreactivity connect the LPC with the medial protocerebrum (Fig. 8d). Strong RFamide-like immunoreactivity is observed in the LPC part of which originated from a large soma located ventrally to the LPC (Fig. 8e), but a differentiation into LPCa and LPCb was not observed with this marker (Fig. 8e).

## Discussion

### Gross brain morphology: differential neural investment into specific subsystems

Abundant studies in the field of arthropod neuroanatomy offer the opportunity to discuss the question as to how the neuronal investment into certain brain regions relates to the habitat, environment or behaviour of an organism (Niven and Laughlin 2008; Chittka and Niven 2009; Harzsch et al. 2012; Sandeman et al. 2014). An emerging consensus suggests that adaptations to the sensory landscape in general are mirrored in the elaboration of sensory pathways and consequently the organization of the primary sensory neuropils, because larger amounts of sensory input necessitate a larger area for neural processing (Chittka and Niven 2009). Consequently, during evolution, specific brain regions may have expanded relative to the rest of the brain in correspondence with ecological specialization. For example, representatives of the genus *Drosophila* demonstrate a differential allocation of neuronal tissues devoted to vision versus olfaction depending on lifestyle (Keesey et al. 2019). Furthermore, well-developed sensory systems and associated brain areas of an animal can be expected to analyze environmental information, which is important and ecologically relevant for the fitness of the organism. For example, analysing the relative neuronal investment into their various brain regions suggests that representatives of shrimps and prawns must rely heavily on processing a dominant mechano- and chemosensory input provided by antenna 2 (Meth et al. 2017). However, considering the high physiological costs of maintaining nervous tissue, we can also expect that poorly used brain areas will quickly diminish during evolution. For example, blind crustaceans from cave or deep-sea environments exhibit reduced visual neuropils but pronounced olfactory pathways reflecting the importance of olfaction in their lightless habitat (Fanenbruck and Harzsch 2005; Ramm and Scholtz 2017; Stegner et al. 2015; Machon et al. 2020).

**Fig. 9:**
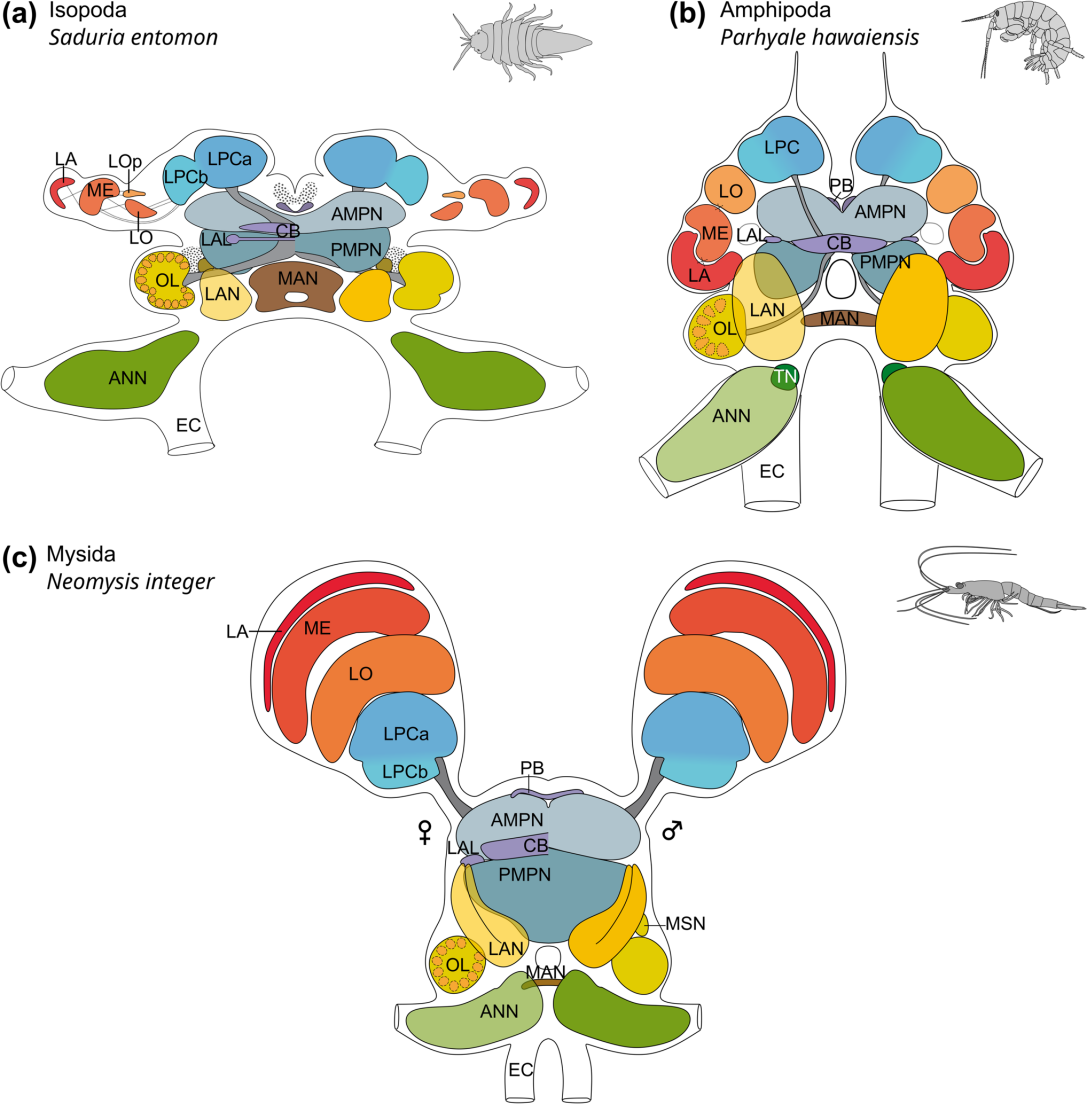
Comparison of brain structure in peracarid crustaceans. **a** Brain schematic of the isopod *Saduria entomon*, modified from Kenning et al. (2013). **b** Brain schematic from the amphipod *Parhyale hawaiensis*, modified from Wittfoth et al. (2019). **c** Brain schematic of the mysid *Neomysis integer*, left half shows female morphology, right side shows male morphology. AMPN, anterior medial protocerebral neuropil; ANN, antenna 2 neuropil; CB, central body; EC, esophageal connective; LA, lamina; LAL, lateral accessory lobe; LAN, lateral antenna 1 neuropil; LO, lobula; Lop, lobular plate; LPC, lateral protocerebrum; LPCa, lateral protocerebrum a; LPCb, lateral protocerebrum b; MAN, medial antennae 1 neuropil; ME, medulla; MSN, male-specific neuropil; OL, olfactory lobe; PB, protocerebral bridge; PMPN, posterior medial protocerebral neuropil; TN, tegumentary neuropil

The morphology of the major brain components in *N. integer* closely corresponds to that proposed for the malacostracan ground pattern (Sandeman et al. 1992, 2014; Kenning et al. 2013). A comparison to other members of the peracarid crustaceans (Fig. 9) suggests that the visual neuropils of *N. integer* are dominant centres in their brains. Their size is considerably larger compared to the olfactory processing regions, indicating that this mysid may depend more on vision than on olfaction. Along these lines, studies mostly on decapod crustaceans have demonstrated that specialized regions in the LPC, medulla terminalis and mushroom bodies receive dominant chemosensory input from the OL *via* the olfactory globular tract in addition to visual and mechanosensory input (reviewed, e.g., in Sandeman et al. 2014; Harzsch and Krieger 2021, 2018), indicating that the LPC functions as a higher, multimodal integration centre. Our data revealed that in *N. integer*, the lateral protocerebrum appeared rather unstructured and inconspicuous, an observation shared with other peracarid representatives, *Saduria entomon* and *Parhyale hawaiensis* (Fig. 9; Kenning and Harzsch 2013; Wittfoth et al. 2019). Given the suggested link between LPC complexity and strength of olfactory input (Sandeman et al. 2014), *N. integer*’s simple LPC reflects its poorly developed olfactory lobe. The CB in the brain of *N. integer* exhibited pronounced immunoreactivity for allatostatin, SIFamide and RFamide, with highly differentiated layer-specific distributions. In hexapods, the central complex receives highly processed visual input and is suggested to function as a higher integrative centre involved in polarization vision, object recognition and motor control of flight and walking, as well as spatial visual memory and place learning (Homberg 2008; Pfeiffer and Homberg 2014; Kandimalla et al. 2023). Although our knowledge about the CB’s functions (including sensory roles) in crustaceans is limited (Strausfeld 2012; Schmidt 2016), the strongly structured and relatively large CB in *N. integer* may correspond to its relatively large visual neuropils, further emphasizing the importance of visual cues.

The antenna 2 neuropil (ANN) located in the tritocerebrum of *N. integer* receives input from the second antenna. Its large size in comparison to other brain areas is notable and likely corresponds to the size of the appendage and number of associated sensilla. In many malacostracans including *N. integer*, the ANN is structured by a striation perpendicular to its long axis, generating the appearance of a stack of discs. Such a pattern is seen, e.g., in the lobster *Homarus americanus* (Sigvardt 1977), the crayfish *Procambarus clarkii* (Tautz and Müller‐Tautz 1983), the banded cleaner shrimp *Stenopus hispidus* (Krieger et al. 2020) and the Pacific White Shrimp *Penaeus vannamei* (Meth et al. 2017). Because the afferents of the bimodal chemo- and mechanosensory sensilla terminating in the ANN display regularly spaced side branches into several of these discs (Tautz and Müller‐Tautz 1983; Schmidt 2016), the striation of the ANN suggests a somatotopic representation of the chemo- and mechanosensory input to this neuropil (e.g., Zeil et al. 1985; Tautz 1987; Sandeman and Varju 1988, reviewed in Schmidt 2007) and consequently a correspondence of the length of this neuropil and the length of the associates antenna 2 (Sandeman et al. 1993; Schmidt 2007). Mellon (2007) emphasized the behavioural importance of processing of hydrodynamic and chemosensory inputs in aquatic crustaceans, and *N. integer* is obviously not an exception here as indicated by its tritocerebral neuroanatomy.

### Sexual dimorphism of the sensillar inventory

The first pair of antennae of *N. integer* is bifurcated and bears diverse sensilla, including a distinct subset termed “slender sensilla” (Johansson and Hallberg 1992a) on both the lateral and medial flagella. Their uniform structure across both flagella indicates they belong to a single sensillar type. While Johansson and Hallberg (1992a) found “slender sensilla” exclusively in male individuals, we observed this type in both sexes. They were suggested to function as bimodal chemo- and mechanosensory sensilla, the most abundant sensilla characterized by terminal pores (Altner et al. 1983; Schmidt and Gnatzy 1984; Cate and Derby 2001, 2002; Hallberg and Skog 2010). Furthermore, malacostracan crustaceans in general possess aesthetascs on the lateral flagellum, which are specialized olfactory sensilla (Hallberg et al. 1992; Schmidt and Ache 1992; Hallberg and Skog 2010; Schmidt and Mellon 2010; Harzsch and Krieger 2018). We identified large, rod-shaped sensilla with strongly autofluorescent tips in *N. integer*. Based on their position and distinctive morphology, and with reference to Hallberg et al. (1992), we propose these structures to be aesthetascs. Nuclear staining further revealed cell clusters located directly beneath these sensilla, which are likely to correspond to the somata of olfactory sensory neurons.

Insects are well known to display a sexual dimorphism in that specialized sensilla transmit sensory input to a male-specific neuropil, the macroglomerular complex (Matsumoto and Hildebrand 1981; Christensen et al. 1995; Zhao et al. 2016; Heinbockel 2022). In contrast, sexual dimorphism in malacostracan crustaceans has not been extensively studied. For example, previous studies showed that male mysids and other male peracarids, such as the amphipod *Hyperia galba*, exhibit structural differences concerning their first pair of antennae compared to females (Hallberg and Skog 2010). Some peracarids possess a specialized region on the first pair of antennae with a high density of aesthetascs, referred to as the “callynophore” (Hallberg and Skog 2010). Another sexual difference involves specific sensilla on the first antenna, as observed in male Amphipoda, Mysida and possibly Cumacea (Hallberg et al. 1992; Hallberg and Skog 2010). Our data also confirm the presence of a sexual dimorphism in the olfactory pathways of *N. integer*. Male individuals exhibit an additional *lobus masculinus* on the first antenna, bearing a multitude of thin, long, simple sensilla, that were previously suggested to represent an adaptation for detecting pheromones released by females (Johansson and Hallberg 1992a). It can be inferred that their high number and great length serve to enhance the probability of binding odorants such as pheromones. This adaptation could be crucial in a reproductive context, given that females are only receptive for a brief period (Clutter 1969; Johansson and Hallberg 1992a; Johansson et al. 1996), and copulation occurs exclusively at night (Johansson and Hallberg 1992a). Consequently, these sensilla may play a significant role in pheromone detection, a hypothesis that warrants further investigation in behavioural studies.

Sexual dimorphism in *N. integer* includes not only male-specific sensilla and the *lobus masculinus* but also unique features in females, such as long, plumose sensilla on the peduncle of the first antenna. While male-specific sensilla have been well-documented, female-specific sensilla are less well studied. We speculate that these plumose sensilla may act as receptors for male pheromone or male-specific metabolic compounds, facilitating intersexual chemical communication, similar to what is known in decapod crustaceans such as *Homarus americanus* and *Callinectes sapidus* (Atema and Steinbach 2007; Kamio and Derby 2011).

### Anterograde fills with neuronal tracers: central brain representation of the input from the first pair of antennae

**Fig. 10:**
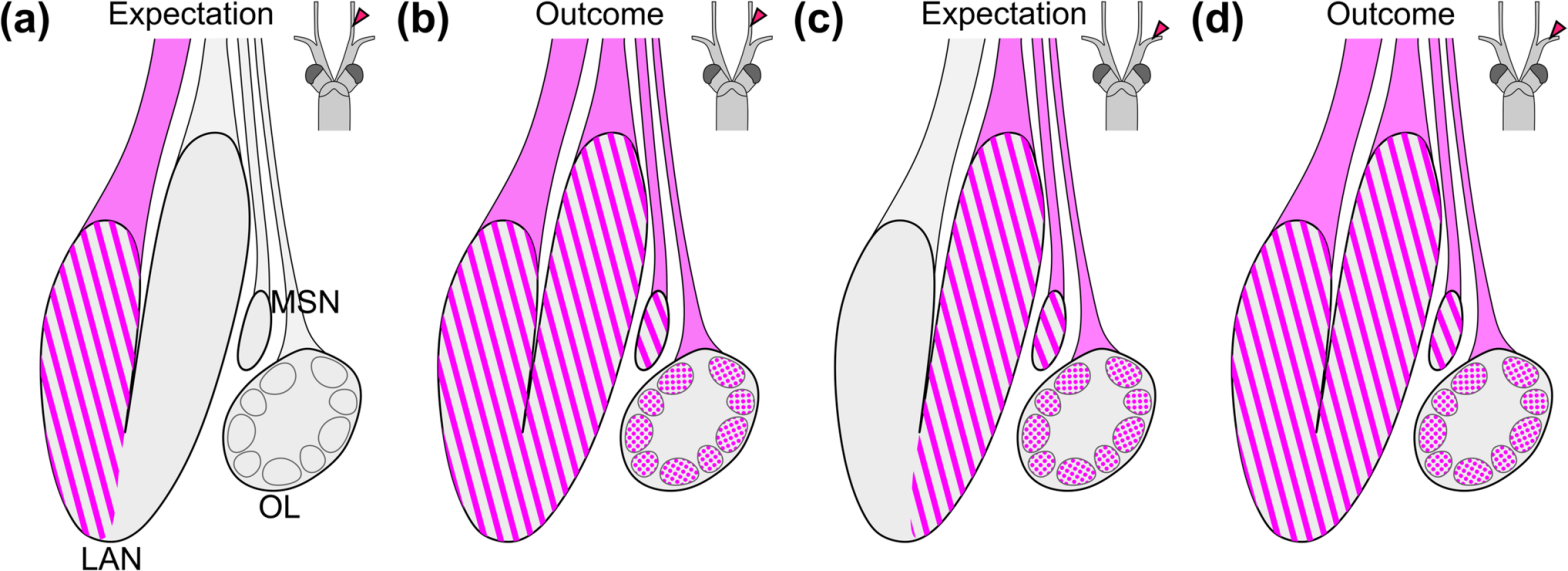
Expectations and outcomes of the first antenna anterograde fills in *N. integer*. **a** Expectation of the outcome of the anterograde fill from the medial flagellum. **b** Outcome of the anterograde fill from the medial flagellum. **c** Expectation of the outcome of the anterograde fill of the lateral flagellum. **d** Outcome of the anterograde fill from the lateral flagellum. LAN, lateral antenna 1 neuropil; MSN, male-specific neuropil; OL, olfactory lobe

To identify brain regions that receive an input from the first pair of antennae, mass anterograde fills were conducted separately targeting the lateral and medial flagella. In decapod crustaceans, the antenna 1 neuropil (LAN) was shown to be primarily innervated by bimodal and mechanosensory sensilla (Tautz and Müller‐Tautz 1983; Schmidt and Ache 1992, 1993, 1996a). The bifurcated structure of the first antenna likely aligns with the bilobed shape of the LAN that has been suggested to display a somatotopic organization (similar to the ANN innervated by the second antenna) so that the spatial arrangement of sensilla in the periphery is topically mapped onto this neuropil retaining the spatial relationship of neighbouring structures (Tautz and Müller‐Tautz 1983; Schmidt and Ache 1996a). Axonal projections from the lateral and medial flagella of the first antennae in spiny lobsters were shown to innervate the lateral and medial LAN, respectively (Schmidt and Ache 1992). Furthermore, the lateral flagellum containing olfactory sensilla projects to the OL in large decapod crustaceans (Schmidt and Ache 1992). Therefore, we expected the lateral flagellum anterograde fill to stain the LAN’s lateral half and the medial anterograde fill to label its medial half (Fig. 10a, c) and anticipated similar outcomes for both sexes. Contrary to our expectations, anterograde fills of either flagellum stained the entire LAN, OL, and in males, the MSN without clear differentiation (Fig. 10b, d). In *N. integer*, the LAN is innervated via dual nerve bundles for each lobe, and cross-innervation may be facilitated by collateral neurites linking the lobes. Furthermore, Johansson and Hallberg (1992a) observed in mysids that the afferent nerve to the LAN bifurcates, converges and subsequently branches again to innervate the lobes of the LAN. This projection pattern may account for our findings, suggesting that neurites from both the lateral and medial flagella may cross over, allowing each lobe of the LAN to receive input from both flagella.

Aesthetascs on the lateral flagellum primarily innervate the OLs, establishing these neuropil’s primary sensory function in olfaction (Schmidt and Ache 1992; Hallberg and Skog 2010; Schmidt and Mellon 2010). However, Schmidt and Mellon (2010) noted that additional chemosensory sensilla, likely bimodal (chemo- and mechanosensory) in function, also contribute to OL innervation in the spiny lobster *Panulirus argus*. Furthermore, in spiny lobsters, a minor subset of mechanosensory afferents may innervate the OL, as indicated by variations in fibre size and branching patterns (Schmidt and Ache 1992, 1996b). This background could explain our observations, where both lateral and medial flagellar anterograde fills labelled the OL, suggesting that bimodal sensilla, in addition to aesthetascs, contribute to OL innervation, thus accounting for OL labelling in medial backfill experiments. Consistent with studies in decapod crustaceans (Schmidt and Ache 1992), we observed thin afferents penetrating the entire OL and innervating distinct olfactory glomeruli.

### The central olfactory pathway: olfactory glomeruli

The axons of olfactory sensory neurons associated with the aesthetascs in malacostracan crustaceans target the OL (Schmidt and Ache 1992; Hallberg and Skog 2010; Schmidt and Mellon 2010). This input is processed within the olfactory glomeruli by a network of local olfactory interneurons (Schachtner et al. 2005; Derby and Weissburg 2014; Harzsch and Krieger 2018). In *N. integer*, the olfactory glomeruli are radially arranged, a pattern also observed in decapods (Harzsch et al. 2022) and other peracarid representatives like amphipods (Kümmerlen et al. 2023) and isopods (Harzsch et al. 2011; Kenning and Harzsch 2013). The number of glomeruli within the OL varies significantly across malacostracan taxa (reviewed in Harzsch and Krieger 2018). Most Peracarida possess less than 100 glomeruli, e.g., the isopod *Saduria entomon* approximately 80 (Kenning and Harzsch 2013) and the amphipod *Parhyale hawaiensis* around 45 glomeruli (Kümmerlen et al., unpublished data). Mysida fall on the lower end of this range having about 35 glomeruli in female and 34 glomeruli in male individuals with a slight variability in number. In decapod crustaceans much higher glomerular numbers occur frequently, reaching in excess of 1000 (Beltz et al. 2003; see discussion Harzsch and Krieger 2018, Kümmerlen et al. 2023). Because a high number of glomeruli likely increases the ability to discriminate odours, the relatively low number of glomeruli in *N. integer* suggests a reduced complexity in their olfactory pathway.

In insects, olfactory glomeruli display individual identities and occur in taxon-specific stereotyped numbers (Stocker et al. 1990; reviewed in Schachtner et al. 2005; Szyszka and Galizia 2015; Fulton et al. 2024). However, evidence for individually identifiable glomeruli in malacostracan crustaceans is missing (Harzsch and Krieger 2018). Despite the numerical simplicity of the mysid central olfactory pathway, our current study nevertheless failed to individually identify the glomeruli and rather provides evidence for some plasticity in glomerular numbers, a feature shared with the amphipod *P. hawaiensis* (Kümmerlen et al., unpublished data).

The local olfactory interneurons innervating the olfactory glomeruli in malacostracan crustaceans display a highly diverse neurochemistry with about a dozen of neuroactive substances identified so far (summarized in Harzsch et al. 2022). In, *N. integer*, we found strong allatostatin-like and RFamide-like immunoreactivity within the olfactory glomeruli, while SIFamide-like immunoreactivity was predominantly localized between the glomeruli. RFamide is a prominent neuropeptide also in the olfactory pathway of decapod crustaceans (Harzsch et al. 2022) and other representatives of the Peracarida, as demonstrated in amphipods (Wittfoth et al. 2019; Kümmerlen et al. 2023) and isopods (Harzsch et al. 2011; Kenning and Harzsch 2013). This neuropeptide has also been located in the brains of mysids, including *N. integer* (Johansson and Hallberg 1992b). Our results confirm and extend their findings and add a description of the distribution of the neuropeptides allatostatin and SIFamide that occurred in a similar pattern as previously reported in the amphipod *P. hawaiensis* (Kümmerlen et al. 2023; Raspe et al. 2023). Although SIFamide immunoreactivity is present in the olfactory projection neurons of the marbled crayfish, *P. virginalis* (Polanska et al. 2007), this neuropeptide did not localize to the projection neurons in *N. integer* and amphipods (Kümmerlen et al. 2023; Raspe et al. 2023), indicating a different functional role in Peracarida. In *N. integer*, the neuropeptides RFamide and allatostatin were localized in granular patches of immunolabelled material in the olfactory glomeruli. In the case of RFamide, the staining co-localized with synapsin, indicating the neuropeptide being present in functional synapses. These granular patches were also observed in the amphipod *P. hawaiensis* where they were suggested to represent complex polyadic synapses or nonsynaptic release sites (Kümmerlen et al. 2023).

### The male-specific neuropil in the central olfactory pathway

 Anterograde filling with neuronal tracers in male *N. integer* revealed a nerve entering the male-specific neuropil (MSN), consistent with the presence of male-specific sensilla on the lateral flagellum and *lobus masculinus* (Johansson and Hallberg 1992a). We showed that the male-specific neuropil was labelled in anteroograde fillling experiments of both medial and lateral flagella, suggesting that, in addition to the *lobus masculinus*, additional male-specific sensilla on the medial flagellum may target this neuropil. Alternatively, this innervation pattern may indicate that the male-specific neuropil integrates broader chemosensory inputs beyond those limited to male-specific sensilla. Our results on anti-synapsin immunoreactivity clearly showed that the MSN is significantly larger than a typical olfactory glomerulus within the olfactory lobe suggesting that it is an important element in the male’s olfactory pathway. Interestingly, the MSN was only detectable using anti-synapsin staining and showed no immunoreactivity for the neuropeptides RFamide, SIFamide and allatostatin. This suggests that none of these analyzed substances are involved in chemosensory processing in the MSN.

It is well-established that in several insect species, male-specific sensilla project their input into an additional neuropil in the deutocerebrum known as the macroglomerular complex (Steinbrecht 1987; Hansson et al. 1991; Christensen et al. 1995). This complex is made up of a system of sex-specific glomeruli and is thought to play a key role in the processing of pheromonal signals (Matsumoto and Hildebrand 1981; Strausfeld and Reisenman 2009). To date, with the exception of mysids (Johansson and Hallberg 1992b), a similar male-specific neuropil has not been identified in any other crustacean. Because in insects, male-specific sensilla innervate the macroglomerular complex, it was suggested that the additional sensilla found in male mysids may serve a similar function in pheromone detection (Johansson and Hallberg 1992a, 1992b; Hallberg et al. 1997). Although physiological evidence for a chemoreceptive role of the *lobus masculinus*, MSN pathway is missing, a chemoreceptive function seems plausible, given the close proximity of the MSN to other chemosensory neuropils and the fact that all chemosensory neuropils in the crustacean deutocerbrum receive input from sensilla on the first antennae (Schmidt and Ache 1992; Hallberg and Skog 2010; Schmidt and Mellon 2010; Harzsch and Krieger 2018). If as in insects the MSN is involved in the detection of pheromones, its large size may indicate an important function in mate search behaviour.

## Data Availability

The data that support the findings of this study are available from the corresponding author upon reasonable request.

## Declarations

### AI Declaration

AI tools were used in certain passages of the text to improve readability.
